# Kikuchi-Fujimoto disease: the quandary continues

**DOI:** 10.1093/jscr/rjab442

**Published:** 2021-10-12

**Authors:** Shannon Caesar-Peterson, Dosuk Yoon, Katrina Tulla, Seyed M Nahidi, Sharang Tickoo, Mudnia Sheikh, Hector Depaz

**Affiliations:** Wyckoff Heights Medical Center, Brooklyn, NY, USA; Wyckoff Heights Medical Center, Brooklyn, NY, USA; Wyckoff Heights Medical Center, Brooklyn, NY, USA; Wyckoff Heights Medical Center, Brooklyn, NY, USA; Wyckoff Heights Medical Center, Brooklyn, NY, USA; Wyckoff Heights Medical Center, Brooklyn, NY, USA; Wyckoff Heights Medical Center, Brooklyn, NY, USA

## Abstract

Kikuchi-Fujimoto disease (KFD) is a rare lymphohistiocytic disorder with an unknown etiopathogenesis. Due to its non-specific lymphadenopathy presentation, treatment is complicated by the frequency by which it is misdiagnosed—for example up to one-third of cases are misdiagnosed as malignant lymphoma, leading to expensive clinical testing and overtreatment of this typically self-limiting illness. KFD has a strong association with SLE, although its transience and rarity make it difficult to investigate. We present a case of KFD to illustrate the variance in presentation and typical outcome of KFD. We want to increase awareness and shed some light on some typical and atypical clinical presentations of KFD to reduce the incidence of misdiagnosis.

## INTRODUCTION

We present a young female who underwent multiple imaging at multiple facilities with no definitive explanation for her axillary pain. An eventual biopsy of a lymph node from the affected area determined the cause to be histiocytic necrotizing lymphadenopathy, known as Kikuchi-Fujimoto disease (KFD) after the first two who initially independently described it. KFD is a rare diagnosis in the USA [3, 4]; however, it is important to realize it as a clinical identity distinct from other etiologies such as malignancy, infection or systemic lupus erythematosus (SLE).

Patients with KFD clinically present with fever and lymphadenopathy, typically in the cervical region. Additional findings can include skin manifestations mimicking SLE, as well as systemic manifestations such as autoimmune hepatitis and aseptic meningitis. Our case presents several variations from the typical presentation in that her lymphadenopathy was limited to the axillary region, sparing the cervical area entirely; her neutrophilia also deviated from the classical presentation of neutropenia in up to 50% of cases [4]. Ultimately, a lymph node biopsy revealed the characteristic focal necrosis of KFD, connected with the need for pathological confirmation of the diagnosis of KFD.

## CASE REPORT

A 41-year-old female presented to our hospital with a 1-month history of swelling and burning pain in the left axilla for which she was seen a week prior and discharged with a NSAID. Additionally, she endorsed a history of fever and night sweats during this period. She appeared generally well and in no acute distress. She denied any radiation of the pain, erythema in the axilla, or lumps in her breast.

**Figure 1:**
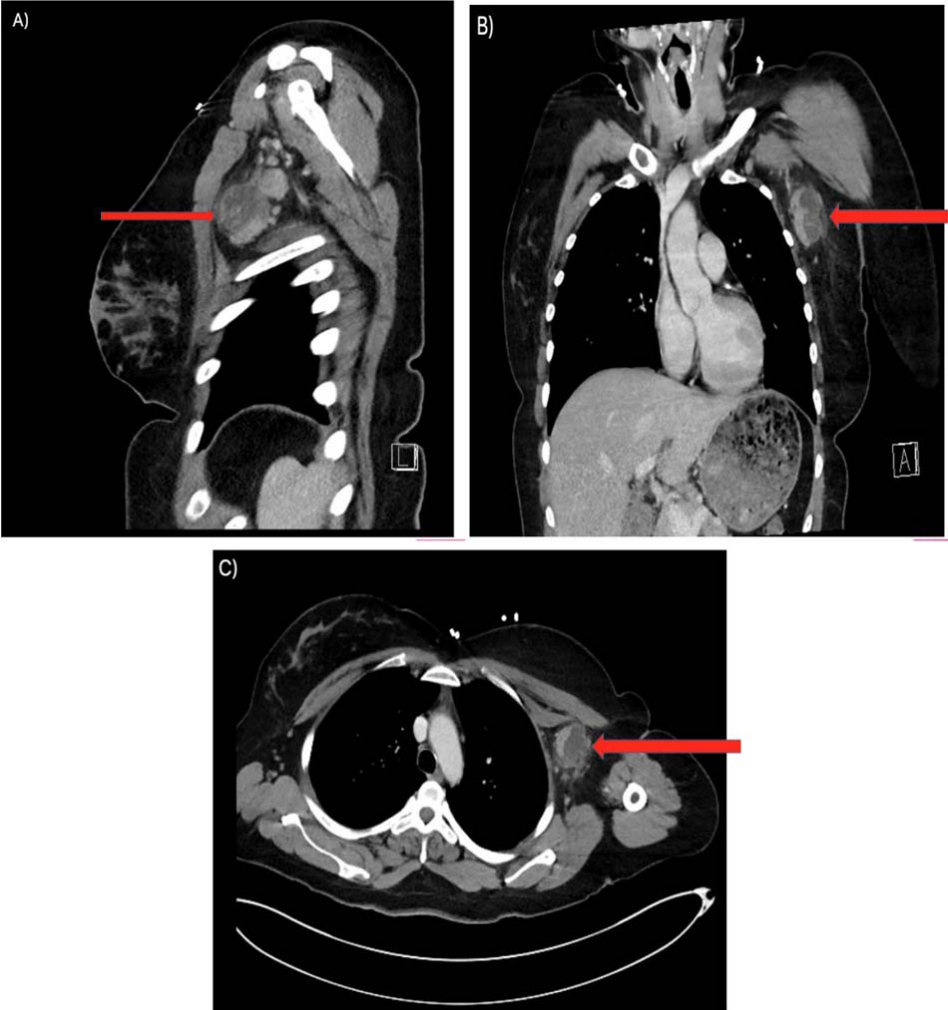
There are multiple enhancing soft tissue lesions involving the left axilla, the largest measuring 4.0 × 3.5 × 4.3 cm and 2.3 × 2.2 × 2.3 cm in maximal AP (**A**), Sagittal (**B**) and Coronal (**C**) Axial, with the largest lesion demonstrating internal necrosis and adjacent infiltrative changes.

**Figure 2:**
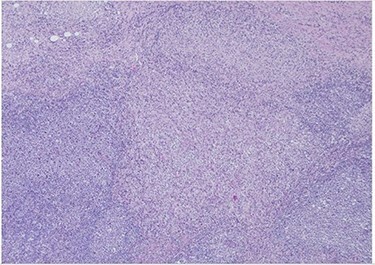
H&E slide (low magnification, 4×) shows characteristic paracortical zonal process involving part of a lymph node. Background consists of preserved nodal architecture with a follicular center and a mantle zone adjacent to intervening pale areas.

**Figure 3:**
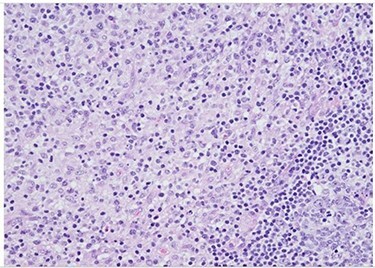
H&E slide (medium magnification, 20×) shows activated lymphocytes, immunoblasts and plasmacytoid monocytes.

**Figure 4:**
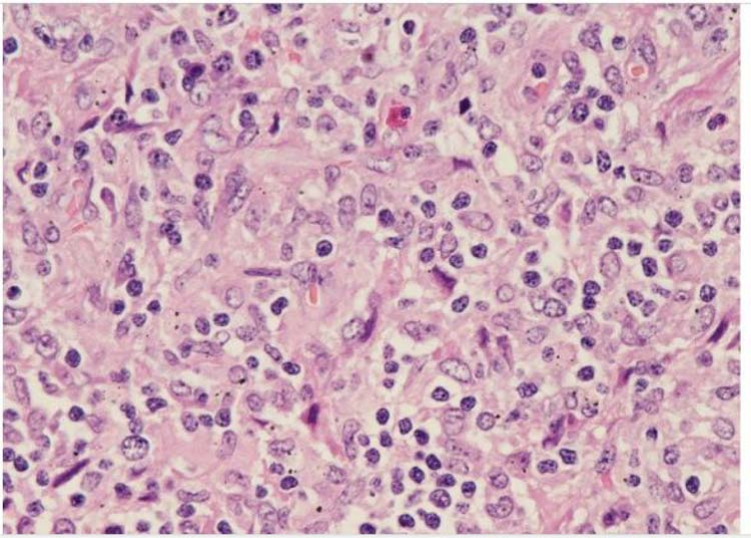
H&E (High power magnification, 40×) shows characteristic large activated lymphoid cells, karyorrhectic debris and histiocytes. The histiocytes are enlarged with crescentic nuclei and phagocytized nuclear debris. Neutrophils are absent.

**Figure 5:**
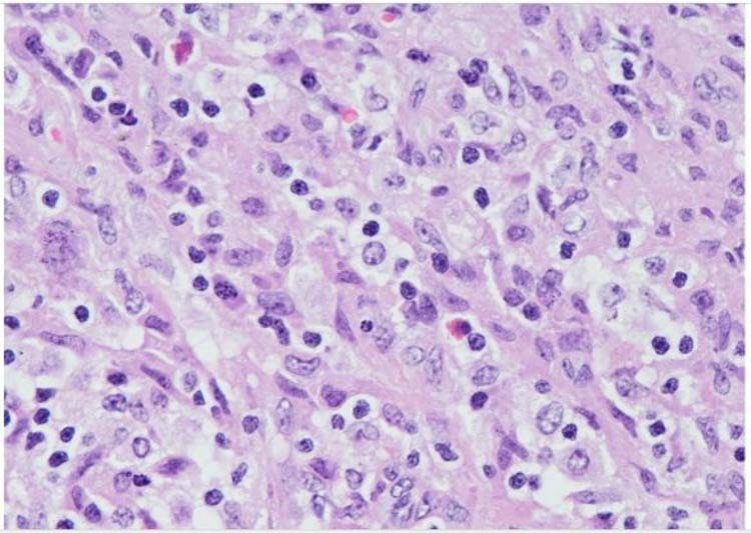
H&E (High power magnification, 40×) shows characteristic large activated lymphoid cells, karyorrhectic debris and histiocytes. The histiocytes are enlarged with crescentic nuclei and phagocytized nuclear debris. Neutrophils are absent.

Her past surgical history included a Cesarean section and internal fixation of her left tibia. Her family history was unremarkable. Her vital signs were all within normal limits. The patient had no significant abnormalities with her routine labs within normal limits except for an elevated WBC count of 18 200 cells/ml. She was tested negative for STIs. The CT scan (Fig. 1) identified multiple enhancing soft tissue lesions involving the left axilla, with the largest lesion demonstrating internal necrosis and adjacent infiltrative changes, while the right axilla was unremarkable. The initial differential included neoplastic, metastatic, infectious or inflammatory processes. General surgery was consulted to rule out an abscess. Discussion was held with the patient about excisional biopsy of the lesion. She had excision of the left axillary mass. The pathology report confirmed the diagnosis of necrotizing histiocytic lymphadenitis (KFD) with focal necrosis devoid of neutrophils in the lymph node (Figs 2–5).

## DISCUSSION

Necrotizing histiocytic lymphadenopathy (KFD), a largely benign and self-limiting illness, was first described independently in 1972 by the Japanese researchers for whom it is named; Kikuchi M and Fujimoto Y [1]. Historically, the disease was most commonly identified in Asian young adults (<40 years of age) with a strong female preponderance [1, 2]. However, cases have been reported across the globe in patients of both sexes and varied ethnic backgrounds [3, 4]. Immunological testing has shown the presence of EBV, CMV and Parvovirus B19 in many cases [5]. Other authors propose auto-immune triggers. In one study, history of SLE was present in 12% of KFD patients, while in other case reports, patients developed SLE after experiencing an incidence of KFD [3, 6]. Overall, a direct mechanistic explanation appears unlikely and the rarity and transience of KFD complicate attempts to elucidate the etiology.

The definitive trait of KFD is lymphadenopathy: most commonly cervical [3]. Mediastinal and axillary nodal involvement is common and there are isolated incidences of retroperitoneal nodal involvement [8]. Pyrexia and neutropenia are additionally present in up to 50% of patients [4]. The most frequently affected extranodal organ is the skin (up to 40% of patients), and in this regard, KFD can resemble SLE due to similar skin manifestations in the form of non-specific rashes, malar erythema, aphthous ulceration, and lupus-like plaques and macules [7, 8].

Due to the varied clinical presentation of KFD, potential systemic complications may require prompt diagnosis and treatment. However, due to the high rate of misdiagnosis and the largely manageable clinical course of KFD, a major concern in these patients is overtreatment for another, more serious illness. Despite the dearth of available literature, there are multiple case reports of initial misdiagnosis of NHL followed by treatment with chemotherapy as far back as the NCI Lymphoma Task Force’s 1973 findings [1]. This is of particular concern when considering the possible simultaneous incidence of neutropenia due to KFD.

Overall, while KFD is a rare illness, it is an important consideration if diagnostic testing for lymphadenopathy does not reveal a clear etiology. Due to the potential for atypical presentations, as with our patient, it is essential to acknowledge that clinical presentation and bloodwork are heuristic tools and not definitive.

## CONCLUSION

KFD has a non-specific clinical presentation that varies from one patient to another. This often results in misdiagnosis or overtreatment of the patient for unrelated illnesses. It is crucial to diagnose or rule out KFD before going through unnecessary testing and treatment, particularly when considering treatment modalities with a high incidence of adverse effects. Since there are no distinctive presentations of this disease, only a biopsy of the abnormal lymph node found on radiological imaging will lead to a conclusive diagnosis.
